# Challenges in Diagnosis and Management of Altered Mental Status in the Setting of Urosepsis and Hydrocephalus Secondary to an Occlusive Cyst of the Fourth Ventricle: A Case Report

**DOI:** 10.5811/cpcem.29335

**Published:** 2025-01-01

**Authors:** Matthew Van Ligten, Miles Hudson, Jonathon J. Parker, Wayne A. Martini

**Affiliations:** * Mayo Clinic, Arizona Department of Emergency Medicine, Phoenix, Arizona; † Mayo Clinic, Arizona Department of Neurosurgery, Phoenix, Arizona

**Keywords:** hydrocephalus, trauma, case report, altered mental status, external ventricular drain

## Abstract

**Introduction:**

Hydrocephalus presents a diagnostic and therapeutic challenge due to its diverse clinical manifestations and underlying causes. Symptoms can vary from feelings of unsteadiness to focal symptoms such as weakness, difficulty ambulating, or urinary incontinence. Due to the wide variety of symptoms, hydrocephalus can present a difficult diagnosis for any physician and may require different interventions depending on the underlying cause.

**Case Report:**

This case report highlights a 69-year-old female with altered mental status, initially diagnosed with communicating hydrocephalus and sepsis. The patient’s symptoms, including confusion, urinary dysfunction, and gait ataxia, initially masked the hydrocephalus, emphasizing the importance of considering this condition in patients with prolonged progression of neurological deficits. Brain imaging, including magnetic resonance imaging (MRI) and computed tomography (CT), facilitated the diagnosis, suggesting hydrocephalus with downward tonsillar herniation. The acute management involved empirical antibiotic therapy for associated sepsis, followed by the placement of an external ventricular drain for cerebrospinal fluid diversion and sampling, including cytology and cell counts, given the concern for tonsillar herniation with a lumbar puncture. Cine MRI and CT cisternogram demonstrated a cyst filling the volume of the fourth ventricle. Subsequent surgical fenestration of the cyst using a suboccipital craniotomy for cyst resection alleviated symptoms and stabilized ventricular size.

**Conclusion:**

Hydrocephalus can present with unique and varying symptoms, and it can have a variety of underlying causes. This case underscores the necessity for individualized treatment approaches tailored to the underlying etiology of hydrocephalus, including temporizing measures and more aggressive approaches once infection has improved.

## INTRODUCTION

Hydrocephalus is defined as the abnormal accumulation of cerebrospinal fluid (CSF) in the cerebral ventricles. Symptoms are widely variable and can occur from a variety of causes ranging from trauma and genetic diseases to infection and autoimmune processes. In the United States, hydrocephalus represents a significant burden to our healthcare system with the average cost of surgical treatments well over $35,000 per intervention. Here, we present a unique case of hydrocephalus in the setting of recent trauma, altered mental status, and sepsis that required advanced imaging to correctly diagnose and treat the hydrocephalus. This case highlights the diagnostic challenge that hydrocephalus can present when an affected patient presents to the emergency department (ED).

## CASE REPORT

A 69-year-old female with a medical history of breast cancer status post mastectomy and hysterectomy presented to the ED for altered mental status after an episode of severe confusion. Upon presentation, the patient stated that she had been feeling progressively dizzy and weak, causing her to fall and strike her head with no loss of consciousness. She also reported difficulty emptying her bladder, dysuria, and two episodes of non-bloody emesis two days prior.

**Figure 1:**
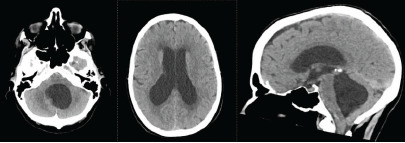
Head computed tomography on admission demonstrating hydrocephalus with enlargement of the lateral ventricles, 3rd ventricle, and 4th ventricle, with downward descent of the cerebellar tonsils.

Vitals were notable for tachycardia and low-grade fever. The physical exam was benign except for mild dysmetria and gait ataxia. Laboratory workup was notable for leukocytosis, a urinalysis consistent with a UTI, normal troponins with no notable change over two hours, and a normal lactate at 1.3 millimoles per liter (mmol/L) (0.5 – 2.2 mmol/L). Basic Metabolic Panel (BMP) showed an elevated serum creatinine at 1.63 mg/dL (0.59 – 1.04 mg/dL) significantly changed from her reported baseline of 0.88 mg/dL. Basic metabolic panel showed signs consistent with dehydration. Chest radiograph revealed no signs of pneumonia, but a computed tomography (CT) head and neck angiogram demonstrated communicating hydrocephalus pattern with dilation of the lateral, third, and fourth ventricles with no signs of intracranial bleeding or skull fracture (Figure 1). The initial differential diagnoses included meningitis, normal-pressure hydrocephalus (NPH), and leptomeningeal tumor spread. Due to the uncertainty, neurology was consulted and recommended a broad infectious workup and magnetic resonance imaging (MRI). Since the patient met criteria for systemic inflammatory response syndrome, she was admitted for further management of her sepsis and hydrocephalus. She was started on vancomycin, ampicillin, and ceftriaxone for empiric meningitis coverage initially, but due to blood cultures growing *Escherichia coli* and the lack of findings concerning meningitis, she was later de-escalated to oral ciprofloxacin.

**CPC-EM Capsule**

What do we already know about this clinical entity?

*Hydrocephalus can present with diverse symptoms, often requiring advanced imaging and individualized treatment*.

What makes this presentation of disease reportable?

*An occlusive fourth ventricle arachnoid cyst, initially occult on imaging, required advanced techniques for diagnosis in the setting of urosepsis and hydrocephalus*.

What is the major learning point?

*Hydrocephalus requires high suspicion in altered mental status cases; advanced imaging may be necessary to diagnose underlying causes that might not be visualized on CT*.

How might this improve emergency medicine practice?

*Recognizing that hydrocephalus may be contributing to altered mental status in patients with sepsis, and using advanced imaging, can prevent misdiagnosis*.

Later imaging via MRI demonstrated slight contrast enhancement of the dura, with the differential including meningitis given her sepsis or leptomeningeal spread of her breast cancer. The MRI also demonstrated periventricular edema with concerns for transependymal flow. These findings were concerning for meningitis vs leptomeningeal tumor spread and communicating hydrocephalus with tonsillar herniation. Given the concern for exacerbation of her downward herniation and crowding of the foramen magnum, a lumbar puncture was deferred, and neurosurgery was consulted. An external ventricular drain (EVD) was placed by the neurosurgical team for CSF diversion and sampling. A full CSF workup was sent, including cultures, cell count, protein, glucose, and cytology. The CSF studies showed a normal opening pressure and no signs of infection, malignancy, or inflammatory process.

**Figure 2:**
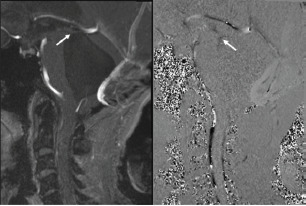
Magnetic resonance imaging brain cine protocol demonstrating obstructed flow near the exit point of the cerebral aqueduct into the 4th ventricle (white arrow).

**Figure 3:**
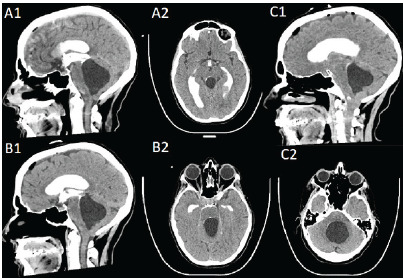
Computed tomography cisternogram. A: 10 minutes after contrast injection demonstrating obstructed flow into the 4th ventricle with some ventral spread of contrast. B: 30 minutes after contrast injection demonstrating further ventral descent of contrast. C: 105 minutes after contrast injection demonstrating ventrolateral spread of contrast to the obex of the 4th ventricle.

Given the negative CSF studies, an MRI with cine protocol was performed to visualize the flow throughout her intracranial ventricular system. The study demonstrated an obstruction at the exit of the aqueduct into the fourth ventricle (Figure 2). A CT cisternogram was performed through the patient’s EVD to further assess the CSF flow dynamics. The study revealed a fourth ventricular cyst obstructing the fourth ventricle, only allowing a small amount of CSF to flow along the right ventrolateral aspect of the cyst (Figure 3). After extensive discussion with the patient, the neurosurgical team recommended a suboccipital craniotomy for fenestration and resection of the cyst to remove the obstruction and decrease the risk of the patient needing placement of a CSF shunt. The procedure was completed without complication, and nine days after admission, her only remaining neurological finding was bilateral nystagmus. An EVD clamp trial was performed, which showed stable ventricular size. The drain was subsequently removed, and she was discharged to a rehab facility for further outpatient management without the need for ventriculoperitoneal (VP) shunting.

## DISCUSSION

Hydrocephalus is a process whereby CSF accumulates inside the cerebral ventricles, causing neurological symptoms that overlap with those of increased intracranial pressure. In adults, there are four distinct types of hydrocephalus: communicating, non-communicating, hypersecretory, and NPH. Common etiologies of communicating hydrocephalus include post-hemorrhagic or post-inflammatory changes, while obstructive hydrocephalus may be secondary to mass-occupying lesions such as tumors or cysts. Normal pressure hydrocephalus is classically defined by the “wet, wobbly, and wacky” triad of urinary incontinence, gait ataxia, and altered mental status. Hypersecretory hydrocephalus is more common in children and due to CSF-producing papillomas or carcinomas.^1^ Regardless of the type, the diagnosis and treatment of hydrocephalus present a challenge to any physician, as the severity and symptoms can range widely and may require advanced imaging, such as the studies used in our case, to successfully treat the underlying reason for the fluid accumulation. This case presented a unique diagnostic challenge as the pathophysiology was obstructive secondary to a large cyst (non-communicating) but presented with a typical communicating hydrocephalus imaging pattern on standard CT and MRI acquisitions.

Given the wide variety of symptoms, hydrocephalus can be found incidentally when investigating a headache, urinary incontinence, or altered mental status in the setting of trauma, as in our patient. Hydrocephalus is often initially diagnosed on CT, but the gold standard is MRI.^2^ Cerebrospinal fluid studies should also be conducted to investigate underlying etiologies for obstruction and hydrocephalus. Lumbar puncture should not be pursued if there is an obstructive hydrocephalus pattern or evidence of downward tonsillar herniation. If initial testing is negative, further imaging, such as an MRI cine, may be useful for diagnosing the underlying cause of the hydrocephalus. For example, MRI cine can be used to differentiate between communicating and non-communicating arachnoid cysts by flow pattern and can be used to gauge response to and monitor for complications of surgical interventions such as VP shunts.^3^ Another useful imaging modality is CT cisternogram, which can be used to accurately diagnose NPH and predict treatment response.^4^ The “tap test” is also an option if imaging is consistent with NPH, where one removes CSF via high-volume lumbar puncture to determine whether a patient’s symptoms improve.^5^

Due to the broad range of possible causes for hydrocephalus, treatment varies widely, with some cases requiring surgical evacuation, VP shunt placement for long-term diversion of CSF, or being treated with resection of the space-occupying lesions. Given this, it is crucial that a physician be aware of advanced imaging techniques such as MRI cine and CT cisternography, which can help elucidate the underlying cause of hydrocephalus and ensure proper treatment. If not identified, temporizing measures such as the EVD that was placed in our case can be done to relieve pressure while investigating possible underlying causes of the CSF buildup.^2^ Outcomes for other interventions, such as ventricular shunts, have also been studied, showing improvement in symptoms in 59% of cases and a 6% severe complication rate.^6^ Today, definitive treatment for hydrocephalus is almost entirely surgical via placement of a shunt, a tappable reservoir to help with serial lumbar punctures, or removal of the obstructing lesion.^7^ Thus, hydrocephalus represents a complex, often puzzling, set of diagnoses that may require further advanced imaging for accurate diagnosis to properly treat the underlying etiology for the accumulation.

## CONCLUSION

Our case demonstrates a unique presentation of a patient with a slow neurological decline from hydrocephalus complicated by an acute worsening due to urosepsis. Further complicating the presentation, the patient’s initial imaging was consistent with a communicating hydrocephalus pattern, which was highly suggestive of meningitis or leptomeningeal tumor spread. As a lumbar puncture was deemed unsafe by neurosurgery, an external ventricular drain was placed for diagnostic and therapeutic purposes to obtain CSF for culture and cytology and to drain CSF. Advanced brain imaging with a cine flow MRI and CT cisternogram confirmed the unique diagnosis of an occlusive fourth ventricular cyst that resulted in a communicating hydrocephalus initial CT pattern in the setting of an obstructive hydrocephalus physiology. This case highlights the need for the emergency physician to look for symptoms of hydrocephalus in patients where lumbar puncture may be considered.
